# Antiviral Treatment Alters the Frequency of Activating and Inhibitory Receptor-Expressing Natural Killer Cells in Chronic Hepatitis B Virus Infected Patients

**DOI:** 10.1155/2012/804043

**Published:** 2012-12-10

**Authors:** Juan Lv, Qinglong Jin, Haibo Sun, Xiumei Chi, Xiaoli Hu, Hongqing Yan, Yu Pan, Weihua Xiao, Zhigang Tian, Jinlin Hou, Damo Xu, Zhengkun Tu, Junqi Niu

**Affiliations:** ^1^Department of Hepatology, The First Hospital, Jilin University, Changchun 130021, China; ^2^Hefei National Laboratory for Physical Sciences at Microscale and School of Life Sciences, University of Science and Technology of China, Hefei 230027, China; ^3^Hepatology Unit and Department of Infectious Diseases, Nanfang Hospital, Southern Medical University, Guangzhou 510515, China; ^4^Institute of Infection, Immunity and Inflammation, University of Glasgow, G12 8TA, UK

## Abstract

Natural killer (NK) cells play a critical role in innate antiviral immunity, but little is known about the impact of antiviral therapy on the frequency of NK cell subsets. To this aim, we performed this longitudinal study to examine the dynamic changes of the frequency of different subsets of NK cells in CHB patients after initiation of tenofovir or adefovir therapy. We found that NK cell numbers and subset distribution differ between CHB patients and normal subjects; furthermore, the association was found between ALT level and CD158b^+^ NK cell in HBV patients. In tenofovir group, the frequency of NK cells increased during the treatment accompanied by downregulated expression of NKG2A and KIR2DL3. In adefovir group, NK cell numbers did not differ during the treatment, but also accompanied by downregulated expression of NKG2A and KIR2DL3. Our results demonstrate that treatment with tenofovir leads to viral load reduction, and correlated with NK cell frequencies in peripheral blood of chronic hepatitis B virus infection. In addition, treatments with both tenofovir and adefovir in chronic HBV infected patients induce a decrease of the frequency of inhibitory receptor^+^ NK cells, which may account for the partial restoration of the function of NK cells in peripheral blood following treatment.

## 1. Introduction 

Chronic infection with hepatitis B virus (HBV) affects more than 350 million people worldwide and continues to be an important cause of morbidity and mortality [1]. The current therapy for chronic hepatitis B (CHB) is based on the use of immunomodulators like pegylated interferon or nucleos(t)ide analogues (NUCs) that inhibit both the priming and the elongation steps of viral DNA replication [2–10].

The high cost, side effects, and the fact that potent antiviral response can be only achieved in a minor population of patients limit the clinical use of PEG-IFN. In addition, as the off-treatment durability of response to NUCs is generally low, it is required to maintain a long-term continuous therapy when patients treated with NUCs. However, long-term continuous therapy with NUCs carries significant risks of occurrence of viral resistance, drug toxicity as well as unsustainable cost for many of the most heavily affected countries. All of these guidelines support both NUCs and PEG-IFN as first-line treatment options, but the optimal choice for individual patients remains controversial. 

The choice of therapy is determined by many factors including the stage of the disease, serum alanine transaminase (ALT), HBV DNA levels, and eAg status of the patient. Both Tenofovir (tenofovir disoproxil fumarate) and Adefovir dipivoxil are very potent and effective nucleotide analog against HBV [9, 11, 12]. But in clinical studies, Tenofovir displayed higher intrinsic potency than Adefovir dipivoxil [12]. Immunologic mechanisms involved in the control of HBV replication in vivo are not yet completely understood [13–17]. Understanding the mechanisms of therapy-induced antiviral immune responses could further direct novel therapeutic strategies [18]. Most studies examined the functionality of HBV-specific CD4^+^ T cells and regulatory T cells in blood of patients and found that their functionality is improved in NUC-treated CHB patients [19–22]. However, this effect was only transient [23]. Besides, HBV-specific T cells also natural killer (NK) are important effector cells during antiviral immune responses [24]. An early rise in circulating NK cells has been documented in the incubation phase of HBV infection, suggesting that they may contribute to the initial viral containment in this setting [25]. NK cells are innate immune cells and play important roles in the defense against viral infections. They can kill virus-infected cells directly as well as indirectly via antibody-dependent, cell-mediated cytotoxicity. They do not need prior sensitization and expansion for this killing. In humans, NK cells are usually defined as CD3^−^CD56^+^ lymphocytes and are comprised of about 5%–20% of peripheral blood lymphocytes. However, the frequency of NK cells in intrahepatic lymphocytes can increase to about 30%–50% [26]. NK cells are a diverse population and NK cells do not possess a single well-defined receptor to recognize antigens on target cells like T cells. Instead, their function depends on the expression of activating and inhibitory receptors that recognize various classes of cell surface ligands [27]. These ligands include classical and nonclassical MHC class I antigens, MHC-like proteins, and a variety of other self- and virus-derived molecules. They may be expressed constitutively and/or de novo on the surface of virus-infected cells.

Only limited information has been published on the role of NK cells in HBV infection. In acute HBV, an early rise in circulating NK cells has been documented, suggesting their contribution to the initial viral containment [25, 28, 29]. In the context of persistent HBV infection, NK cell studies mainly focused on NK cell induced tissue injury [30, 31]. However, very little is known about the quality of the antiviral functions of NK cells during chronic HBV. It has recently shown that inhibition of chronic hepatitis B virus replication by antiviral therapeutic medicine such as entecavir, lamivudine, and adefovir helps to partially restore function of NK cells in peripheral blood [32, 33]. This restoration of NK cell activity was accompanied by an enhanced frequency of IFN-*γ*-producing CD56^+^ NK cells in blood as well as normalization of the expression of the activating receptor NKG2A on circulating NK cells. Controlling of viral replication by antiviral treatment can partially correct this defect but little is known about the impact of antiviral therapy on the frequency of different subsets of NK cells. To this aim, we performed this longitudinal study to examine the dynamic changes of the frequency of different subsets of NK cells in CHB patients after initiation of tenofovir or adefovir therapy. We found that NK cell numbers and subset distribution differ between CHB patients and normal subjects, furthermore the association was found between ALT level and CD158b^+^ NK cell in HBV patients. In CHB patients treatment with tenofovir, the frequency of NK cells was increased during the treatment, accompanied downregulated expression of NKG2A and KIR2DL3, which correlated with serum HBV-DNA load. In CHB patients treatment with adefovir, NK cell numbers did not differ during the treatment, but also accompanied downregulated expression of NKG2A and KIR2DL3.

## 2. Patients and Methods 

### 2.1. Patients and Healthy Subjects

A total of 24 CHB patients and 12 gender- and age-matched healthy subjects were recruited at the outpatient clinic of the Department of Hepatology of the First Hospital of Jilin University, China from May 2011 to October 2011. Individual patients with CHB were diagnosed, according to the criteria of detectable HBV virions and HBeAg positive or negative (HBVDNA ≥ 105 copies/mL, an interval of 14 days or more than twice elevated ALT of HBeAg-positive patients ≥ 2 ULN(upper limit of normal); HBeAg-negative patients > 1 ULN and ≤ 10 ULN). There are seven HBeAg-positive patients in TDF-group and eight in ADV-group. All the 24 patients were previously untreated. Individuals with a history of hepatitis C and hepatitis D, positive antibodies against HCV and HDV, human Immunodeficiency virus (HIV), or other inflammatory diseases, such as rheumatoid arthritis, diabetes, autoimmune hepatitis, hypertension, kidney disease, or recent infectious diseases were excluded. Written informed consent was obtained from all patients, and the experimental protocol was approved by the Ethics Committee of the First Hospital of Jilin University.

### 2.2. Treatment

Individual patients with CHB were randomized and treated with tenofovir disoproxil fumarae (GSK Pharma UK) 300 mg once daily. Another group of patients with CHB were treated with adefovir disoproxil (GSK Pharma UK) 10 mg once daily. All of the patients met the most recent European guideline criteria for treatment of CHB [34] and were followed up for 24 weeks. Peripheral EDTA anticoagulant blood samples were obtained from individual participants before and after the treatment longitudinally. 

### 2.3. Viral Genotyping, Viral Load and Other Biochemi cal Measurements

HBVserology (HbsAg/Anti-HBs,HbeAg/Anti-Hbe,quantitative HbsAg, quantitative HbeAg) were determined by microparticle enzyme immunoassay (MEIA). The virus loads in individual plasma samples were measured by quantitative PCR using Roche COBAS Taqman HBV test (Roche, UK), and the limitation of detecting HBV was 20 IU/mL.The levels of serum aspartate aminotransferase (AST) and alanine transaminase (ALT) were detected by Biochemistry Automatic Analyzer (Roche Diagnostics, Branchburg, USA). 

### 2.4. Flow Cytometry Analysis

To determine the percentages of different subsets of NK cells, anticoagulated blood was incubated for 30 min with a cocktail of allophycocyanin (APC)-conjugated CD56 (clone B159), fluorescence isothiocyanate (FITC)-conjugated CD3 (clone UCHT1), and PerCP-Cy 5.5-conjugated CD16 (clone 3G8), and stained with PE-conjugated antibodies against CD158a (clone HP-3E4), CD158b (clone CH-L), NKp30 (clone P30-15), NKp44 (clone p44-8.1), NKp46 (clone 9E2/Nkp46, BD PharMinge, San Diego, USA), NKB1 (clone DX9, BD Biosciences, Belgium), KIR2DL3 (clone 180701), NKG2D-PE (clone 149810), NKG2A (clone 131411), NKG2C (clone 134591, R&D Systems, USA), or isotope controls, respectively. The frequency of different subsets of NK cells was characterized by flow cytometry analysis. 

### 2.5. Statistical Analysis

Data are expressed as mean values of individual patients or median and range. The difference between two independent groups was analyzed by the Mann-Whitney *U* test, and the difference between paired variables by the Wilcoxon matched-pairs test using the Prism 5.0 (GraphPad software, USA). The potential correlation between two variables was analyzed by the Spearman's rank correlation coefficient test. A two-tailed *P* value of less than 0.05 was considered statistically significant. 

## 3. Result

### 3.1. Alteration in the Frequency of NK Cells Is Associated with Liver Damage in CHB Patients

To determine the frequency of NK cells, 24 CHB patients and 12 healthy age and gender matched subjects were recruited into this study. CHB patients displayed high concentrations of ALT and HBV loads (Table 1).

**Figure 1:**
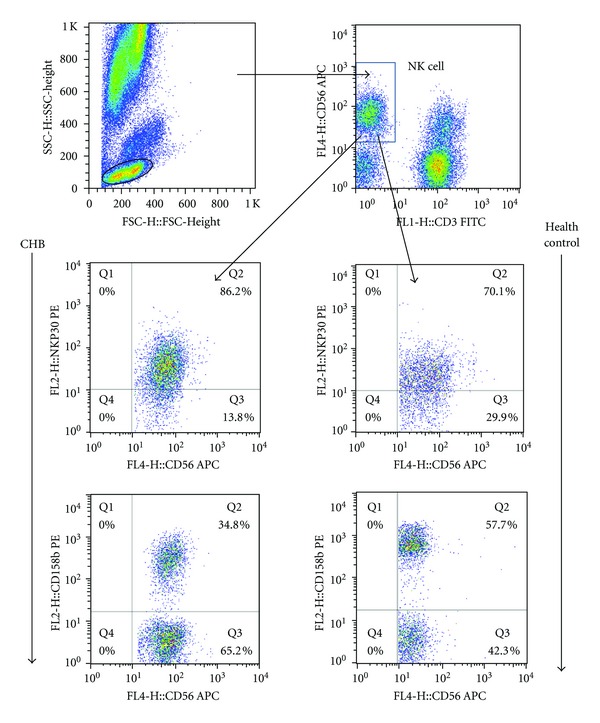
Characterization of different subsets of NK cells. The frequency of different subsets of NK cells was determined by flow cytometry analysis using specific antibodies. Briefly, peripheral blood mononuclear cells were prepared from individual subjects and stained with a cocktail of APC-anti-CD56 and FITC-anti-CD3 and the cells were gated on CD3^−^CD56^+^ as NK cells. Furthermore, the cells were stained with APC-anti-CD56 and PE-conjugated antibodies against CD158a, or CD158b, NKp30, NKp44, NKp46, NKB1, KIR2DL3, NKG2D, NKG2A, NKG2C, or isotope controls, respectively, for characterizing the frequency of different subsets of NK cells. Data shown are representative charts of flow cytometry analysis of some subsets of NK cells.

**Figure 2:**
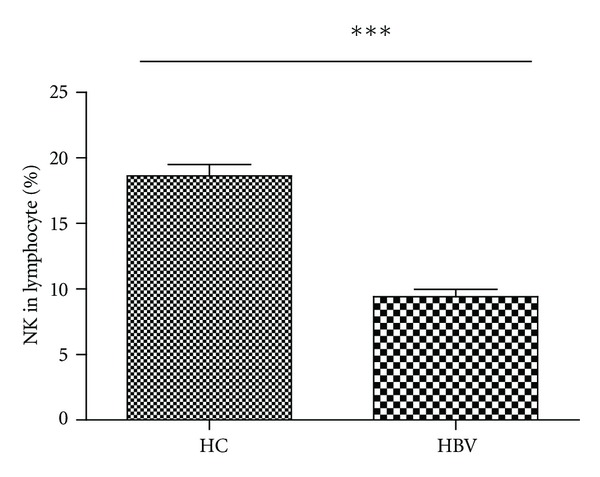
Characterization of NK cells at baseline. The percentages of NK cells in lymphocytes. Data are expressed as mean ± SEM. ***P < 0.001.

**Figure 3:**
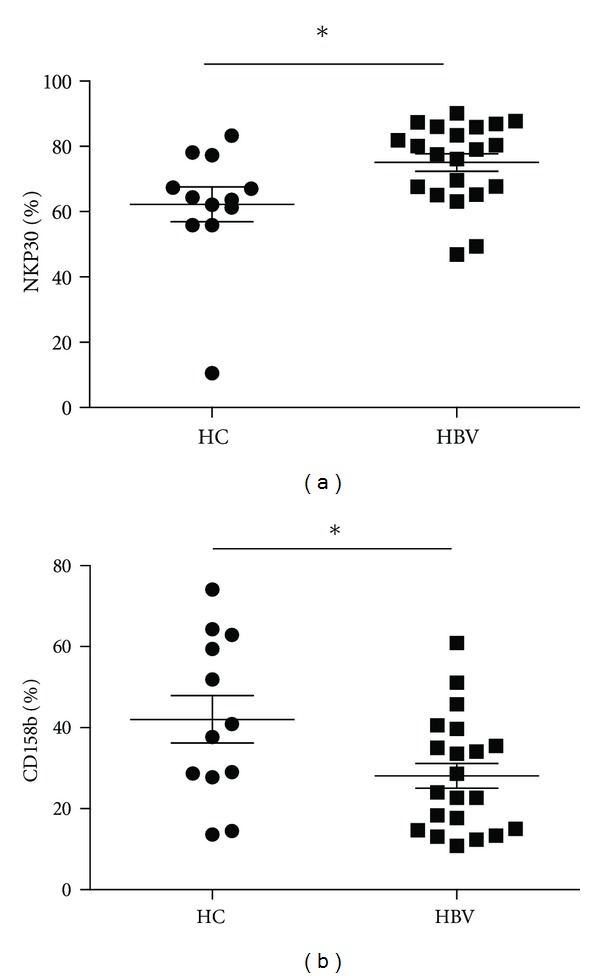
Expression of activating and inhibitory receptors on NK cells in patients with chronic HBV infection. The frequency of activating receptors NKp30 and inhibitory receptors CD158b on NK cells in peripheral blood of 12 healthy controls (HC) and 21 sex and age-matched chronic HBV patients (HBV) (mean ± SEM HBV-DNA 10log 7.5 ± 0.9 IU/mL, ALT 167 ± 96 IU/mL, 9 e-negative Ag) was determined by FACS analysis. Data show the individual and the mean percentage of expressing cells within the total NK cell population. *P < 0.05.

**Figure 4:**
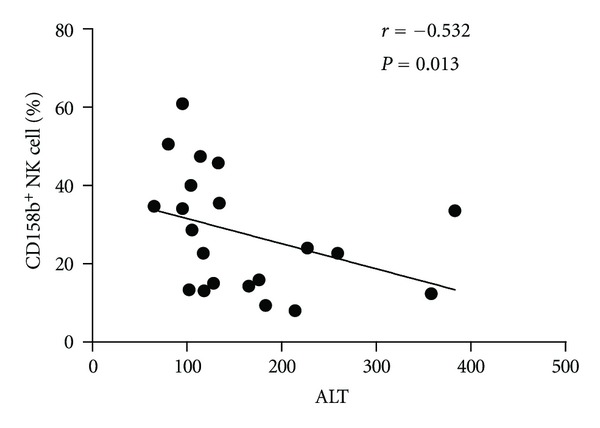
Liver injury was negatively correlated with inhibitory receptor CD158b expression on NK cells. The potential correlation of the percentages of NK cells with the levels of serum ALT in CHB patients was analyzed.

Characterization of peripheral blood NK cells by flow cytometry analysis indicated that the frequency of CD3^−^CD56^+^ NK cells in CHB patients was lower than that in healthy controls (Figures 1 and 2). Furthermore, analysis of different subsets of NK cells revealed that the frequency of activating receptor NKp30^+^ NK cells in CHB patients was significantly higher than that in healthy controls while the frequency of inhibitory receptor CD158b^+^ NK cells in CHB patients was found to be significantly lower than that in healthy controls(Figures 1 and 3). We observed no significant difference in the expression of NKp44, NKp46, NKG2A, NKG2C, NKG2D, NKB1, and CD158a between the health controls and CHB patients. Interestingly, the concentrations of plasma ALT in CHB patients were negatively associated with the frequency of CD158b^+^ NK cells in CHB patients (Figure 4). These data indicated that alteration in the frequency of NK cells was associated with liver damage in CHB patients. 

### 3.2. Patients Have Different Therapeutic Response to TDF and ADV Treatment

These patients were treated with tenofovir or adefovir. In patients treated with tenofovir or adefovir, ALT, AST, and HBV DNA were markedly reduced after initiation of therapy. There were 7 patients (rate = 58%) whose HBV DNA reaches <3log10 IU/mL in the TDF treated group while only one patient's HBV DNA reaches this level in ADV treated group (rate = 8.3%) (Table 2). The viral response rate of TDF-group (HBV DNA < 3log10 IU/mL) was significantly higher than ADV-group at 8 weeks (*P* = 0.028, Fisher test). In seven HBeAg-positive patients at 24 weeks after treatment of Tenofovir disoproxil, three cases achieved HBeAg seroconversion. However, no HBeAg seroconversion occurred in 8 patients with adefovir dipivoxil treatment. Therefore, different treatments may yield different responses, TDF at a daily dose of 300 mg had superior antiviral efficacy compared with ADV at a daily dose of 10 mg through week 24. 

### 3.3. Treatment with Tenofovir or Adefovir Alters the Frequency of Different Subsets of NK Cells in CHB Patients

**Figure 5:**
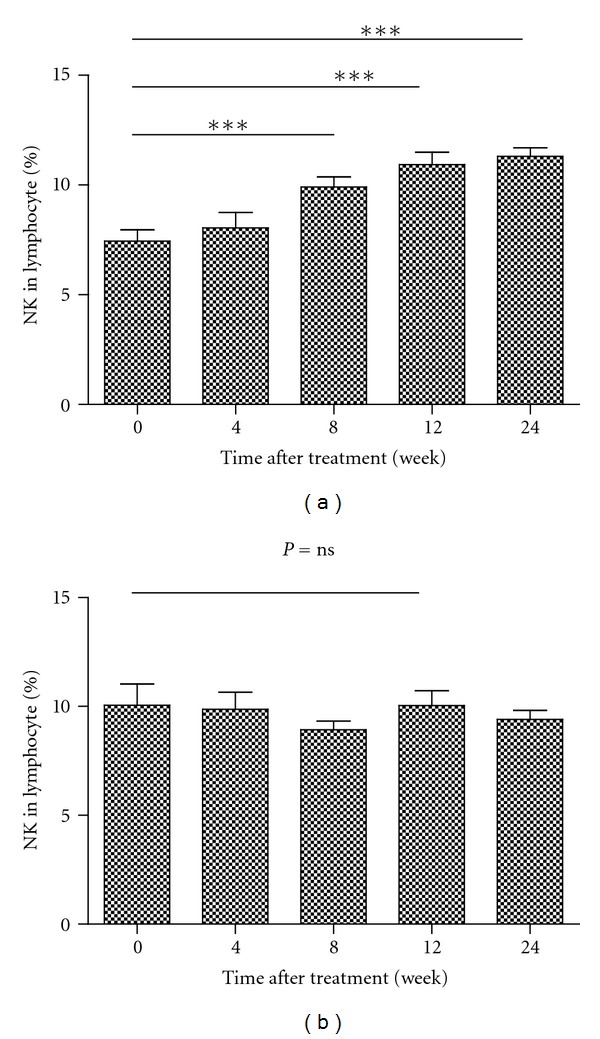
Antiviral treatment TDF but not ADV results in increase of NK cells. Data are presented as mean ± SEM (a). **P < 0.01, ***P < 0.001 Data are expressed as mean ± SD of the percentages of NK in 12 patients treated with tenofovir (a) or adefovir (b) at the indicated time points after treatment. (a, b): the percentages of NK cells in lymphocytes.

We next examined the impact of different treatments on the changes of NK cells. We found that the frequency of NK cells transiently increased in tenofovir treated group at 8, 12, and 24 weeks after initial treatment (Figure 5). These data clearly indicated that treatment with tenofovir modulated the frequency of NK cells. 

**Figure 6:**
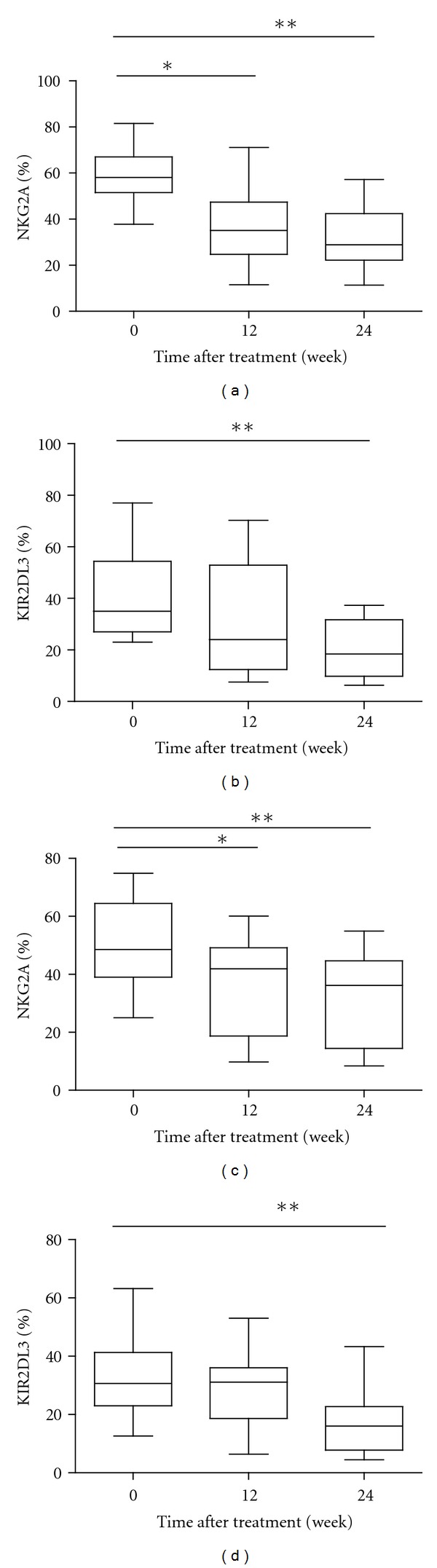
Antiviral treatments results in decrease of NKG2A and KIR2DL3 expression in patients with chronic HBV infection treated with tenofovir or adefovir. The frequency of NKG2A and KIR2DL3 expressing NK cells at baseline (0w), 12 w, and at 24 w of therapy. Data are shown in box-and-whisker plots (a)–(d). (TDF-group (a) (b)): changes in the frequency of inhibitory receptor^+^ NK cells; (ADV-group (c) (d)): changes in the frequency of inhibitory receptor^+^ NK cells.

**Figure 7:**
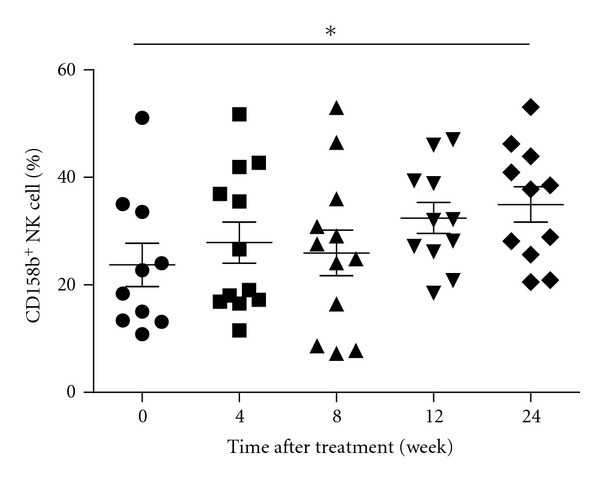
The effect of treatment with ADV on the frequency of CD158b^+^ NK cells in CHB patients. Data are expressed as mean values of individual patients at the indicated time points after treatment.

Given that different subsets of NK cells have different functions, we characterized the frequency of different subsets of NK cells longitudinally after treatment. Treatment with tenofovir or adefovir decreased the frequency of NKG2A^+^ and KIR2DL3^+^ NK cells at 12 and 24 or 24 weeks after initial treatment in both groups of CHB patients (Figure 6), respectively. In contrast, treatment with adefovir significantly increased the frequency of CD158b^+^ NK cells at 24 weeks after initial treatment in CHB patients (Figure 7). 

 Therefore, treatment with different kinds of nucleos(t)ide analogues (NUCs) had differential effects on modulating activating and inhibitory receptor^+^ NK cells in CHB patients.

## 4. Discussion 

In this study, we examined the frequency of different subsets of NK cells in patients with CHB following nucleos(t)ide analogues (NUCs) antiviral therapy. We found that, there was significant difference in the frequency of NK cells between CHB patients and healthy controls, the frequency of NKp30^+^ NK cells in CHB patients was significantly higher than those in healthy controls. In contrast, the frequency of CD158b^+^ NK cells in CHB patients was significantly lower than that in healthy controls. More interestingly, the concentrations of serum ALT were negatively correlated with the percentages of CD158b^+^ NK cells in CHB patients. These data indicated that a lower frequency of inhibitory receptor^+^ NK cells contributed to liver damages. Apparently, a lower frequency of CD158b^+^ NK cells may be valuable for the evaluation of liver damage in CHB patients. 

Previous studies have shown controversial results on the frequency of NK cells in CHB patients following standard therapies [33, 35]. We treated CHB patients with tenofovir or adefovir and characterized the frequency of different subsets of NK cells by flow cytometry analysis. We found that treatment with tenofovir transiently increased the frequency of NK cells in CHB patients at 4, 12, and 24 weeks after initiation of treatment. In contract, the frequency of NK cells does not change significantly over the course of treatment with adefovir. These data suggest that treatment with tenofovir may promote NK cell proliferation during the early periods after treatment. Maybe because of TDF had superior antiviral efficacy compared with ADV. 

Characterization of different subsets of NK cells indicated that treatment with the antiviral therapy transiently decreased the frequency of NKG2A^+^ and KIR2DL3^+^ NK cells after treatment. More importantly, the decreased percentages of NKG2A^+^ NK cells were negatively correlated with the reduced levels of HBV loads in CHB patients early after treatment. These data were similar to that of previous reports in chronic hepatitis B virus infected and chronic hepatitis C virus infected patients [32, 36]. Although we could not establish significant correlations between the expression of NK cell receptors, such as NKG2A, KIR2DL3, and activation status of function, it does not exclude a role for these receptors in the regulation of NK cell activation and/or function. Since NKG2A triggering have been shown to regulate IFN-*γ* production [37, 38], it is tempting to speculate that in chronic HBV infection, increased expression of NKG2A prohibits NK cells from being activated and from functioning. In that respect, the downregulation of NKG2A upon viral load reduction as demonstrated herein may be in part responsible for the improved NK cell function. These data suggest that NKG2A^+^ NK cells are crucial for the clearance of HBV in CHB patients and that the frequency of peripheral blood NKG2A^+^ NK cells may be a valuable biomarker for the evaluation of HBV clearance in CHB patients. 

The balance of activating signals via NCR and inhibitory signals via KIR is crucial for systemic NK cell responses, and HBV infection can modulate the interplay between inhibitory and activation signals [39, 40]. To further understand the mechanisms of therapy-induced antiviral immune responses, we characterized the frequency of different subsets of NK cells in CHB patients with different NUCs treatments. We found that treatment with either protocol decreased the frequency of NKG2A^+^, KIR2DL3^+^NK cells later after treatment. Apparently, treatment with NUCs down-regulates inhibitory receptor expression in NK cells in CHB patients. These data further indicate that downregulates inhibitory receptor expression in NK cells which can increase NK activity is associated with the clearance of HBV in CHB patients. Therefore, given that treatment with antiviral therapy significantly reduced the levels of plasma HBV loads and serum ALT, the higher frequency of NK cells and decreased inhibitory receptors suggested that activated NK cells participated in the clearance of HBV in CHB patients. We compared the different subsets between tenofovir group and adefovir group, adefovir promoted the percentage of CD158b^+^ NK cells. The frequency of CD158b^+^ NK cells significantly correlates with the ALT level in CHB patients before initiation of antiviral treatment. The increased percentage of CD158b^+^ NK cells may be associated with the decreased concentrations of plasma ALT. 

Several groups have studied NK cells during the antiviral treatment of CHB patients, but the results regarding NK cell frequency and receptor expression are conflicting [32, 40, 41]. This probably reflects the complexity of activating and inhibiting signals that control NK cells. One important question is how the responses of individual patients to the antiviral therapy are associated with the change in the frequency of different subsets of NK cells. We noted a rapid decline in expression of NKG2A and KIR2DL3, but not activating receptors, following the administration of NUCs. This implies that these cells have this balance altered in favour of activation. Viral load reduction may be an indirect effect on NK cells due to changes in the cytokine milieu, or due to interactions with other cells of the immune system that can subsequently effect NK cell receptor expression. During the treatment of CHC patients, the frequency of NKG2A^+^ and KIR2DL3^+^ NK cells in the EVR group was significantly higher than those in the non-EVR group [42]. Interestingly, a recent study has shown that KIR2DL3^+^ NK cells have higher degranulation activity in patients with self-limited HCV infection [43]. Different from hepatitis C treatment, besides the decrease of viral load, HbeAg seroconversion was also an important standard to judge the antiviral effect. In this study, subjects of HbeAg seroconversion were limited to analysing the difference frequency of NKG2A^+^ and KIR2DL3^+^ NK cells bewteen HbeAg seroconversion and none-HbeAg seroconversion at 24 weeks after initial treatment. We will analyse it in the followup.

Therefore, the frequency of NKG2A^+^ and KIR2DL3^+^ NK cells may be a prognostic marker for the evaluation of individual responses to the standard therapy in the clinic. 

In summary, our data indicate that the frequency of CD3^−^CD56^+^ NK cells in CHB patients was lower than that in healthy controls, the frequency of NKp30^+^NK cells in CHB patients was significantly higher than those in healthy controls. In contrast, the frequency of CD158b^+^ NK cells in CHB patients was significantly lower than that in healthy controls. The percentages of inhibitory CD158b^+^ NK cells were negatively associated with liver damage in CHB patients. Treatment with the tenofovir therapy transiently increased the frequency of NK and decreased frequency of inhibitory receptor^+^ NK cells in CHB patients. Therefore, our findings provide a unique insight in the NK cell compartment in CHB patients during tenofovir induced viral load reduction. Our study demonstrates modest changes in the frequency of inhibitory receptor^+^ NK cells after successful antiviral therapy. We recognized that our study had limitations of small sample size and the lack of analysis of NK cells function and NK cells in the target tissue. Thus, further studies with a large population, combined with liver tissue analysis, NK cells function, to validate the findings are warranted.

## Figures and Tables

**Table 1:** Characteristics of study population.

	Healthy controls (n = 12)	ADV-group (baseline, n = 12)	TDF-group (baseline, n = 12)
Age, years (mean ± SEM)	35.9 ± 10.1	33.5 ± 5.9	35.2 ± 6.44
Sex			
Female/male (%)	2/10 (17/83)	2/10 (17/83)	5/7 (42/58)
ALT, IU/mL (median (IQ range))	n.a	125.5 (65–383)	123.5 (95–358)
HBV-DNA mean log⁡⁡10 IU/mL ± SEM	n.a	7.7 ± 0.9	7.5 ± 1.0
HBeAg			
Positive/negative	n.a	8/4	7/5
HBV genotype			
Genotype B/C		2/10	0/12

Abbreviations: SEM: standard error of mean, IQ: interquartile, n.a.: not applicable.

**Table 2:** Virological outcomes to tenofovir or adefovir.

Time point	TDF	ADV
Baseline	8.01 (6.22–9.22)	7.81 (5.89–8.86)
4 weeks	5.16 (2.91–6.77)*	4.58 (2.79–6.33)*
8 weeks	2.95 (2.34–5.98)*	3.58 (1.89–5.96)^∗#^
12 weeks	2.42 (1.62–5.47)*	2.83 (1.88–5.63)*
24 weeks	1.85 (1.3–4.8)*	2.21 (1.3–5.57)*

Compare with the initial of the treatment, *P < 0.05; compare with two drugs, ^#^ *P* < 0.05.
